# Stress, repetitive negative thinking, and mental health in Chilean university students: an ecological momentary assessment study

**DOI:** 10.3389/fpsyg.2024.1400013

**Published:** 2024-07-19

**Authors:** Carolina Inostroza, Claudio Bustos, Vasily Bühring, Lorena González, Félix Cova

**Affiliations:** ^1^Departamento de Psicología, Facultad de Ciencias Sociales, Universidad de Concepción, Concepción, Chile; ^2^Programa de Magíster en Psicología, Facultad de Ciencias Sociales, Universidad de Concepción, Concepción, Chile

**Keywords:** Stress, repetitive thinking, rumination, mental health, university students, ecological momentary assessment

## Abstract

**Background:**

Mental disorders in university students are a growing attention problem in the international community due to their high prevalence and serious consequences. One possible reason is university students’ difficulties in coping with stress. Repetitive negative thinking (RNT) is a transdiagnostic process that, when combined with stress, can lead to the development of various disorders. We aim to determine the effect of stress and RNT on predicting various mental health syndromes in university students across 7 days.

**Method:**

Prospective observational study using Momentary Ecological Assessment (EMA) with the OURMIND Mobile App. On day one, 238 university students responded to the SCL-90R questionnaire for symptoms of depression, anxiety, hostility, obsession, psychoticism, paranoia, somatization, and interpersonal sensitivity; RNT styles questionnaires, RRS for rumination and negative reflection, PSWQ for worry; SISCO-II for term academic stress, and sociodemographic. EMA consisted of five assessments a day for 6 days; each time, the students answered items about academic and non-academic stress (EMA-stress), reactive RNT duration and intrusiveness (EMA-RNT process), and reactive RNT rumination, reflection, and worry (EMA-RNT content). On day eight, symptoms were re-assessed. Seven hierarchical stepwise linear regression models were used to test the predictive power of the study variables in the development of SCL-90R symptoms.

**Results:**

When comparing models, adding baseline symptoms increased the models’ predictive power in all symptom groups. In most cases, including EMA-stress generated greater predictive power, except for paranoia and interpersonal sensitivity. Adding the EMA-RNT process increased the prediction of paranoia and obsessive symptoms; for hostility symptoms, RNT styles increased predictive power. For the final regression models, considering the initial symptoms, the EMA-RNT process predicted the progression of symptoms in six out of eight groups, while EMA-non-academic stress predicted the remaining two. Additionally, living with other relatives or friends was a predictor of depressive symptoms.

**Discussion:**

The stress of university life impacts the development of psychiatric symptoms in university students. These results provide evidence of RNT as a transdiagnostic process in several syndromic groups. Universal preventive programs should consider the impact of academic and non-academic stress on university students’ mental health. Targeting RNT would also benefit selective preventive interventions.

## 1 Introduction

The mental health of university students is a problem of growing attention in the international and national community due to the high prevalence of disorders and their serious consequences (Auerbach et al., 2018; Mac-Ginty et al., 2021), including suicide, the second cause of death in this age range (World Health Organization, 2014; Turecki et al., 2019). In Chile, reviews of prevalence studies in university students have estimated a range from 22.9 to 40.7% for psychological distress (Martínez et al., 2021). According to a multicenter study conducted by the World Mental Health International College Student Initiative (WMH-ICS) (Auerbach et al., 2018), 31% of college students experienced some form of mental disorder in the past year.

Longitudinal studies show that mental disorders usually occur in youth and tend to stabilize in adult life. Follow-up studies show that anxiety and depression disorders at these ages are still present ten years later (Gustavson et al., 2018), which highlights the relevance of conducting studies in this population.

### 1.1 Transdiagnostic approach

The transdiagnostic approach stands out within the comprehensive models for mental disorders developing in recent decades (Barlow et al., 2004; Harvey et al., 2004; Ehring and Watkins, 2008). This approach emerges as a response to the deficits of categorical classification systems (Sandín et al., 2012; Dalgleish et al., 2020). It posits that mental problems are caused by processes that exist in the population on a gradient from normal to dysfunctional and that these psychopathological processes are common to more than one disorder, which would explain the high comorbidity and transitions between diagnoses across the lifespan (Nolen-Hoeksema and Watkins, 2011; Pascual-Vera and Belloch, 2018). The transdiagnostic approach gains increasing support from psychiatry and psychology thanks to advances in research on psychopathological processes and the development of effective psychotherapeutic intervention programs (Norton and Paulus, 2016; Pascual-Vera and Belloch, 2018; Heckendorf et al., 2019; Dalgleish et al., 2020; Eustis et al., 2020; Leonardo et al., 2021).

Transdiagnostic processes correspond to nuclear dysfunctional processes that are shared by different mental disorders with high comorbidity, which can be explained by similar theoretical models and respond to the same psychotherapies or treatments (Pascual-Vera and Belloch, 2018; Dalgleish et al., 2020). After decades of research in this area, Nolen-Hoeksema and Watkins (2011) pointed out that further investigation of certain elements of the transdiagnostic model is required. One of them is the multifinality of the transdiagnostic process, i.e., how a transdiagnostic process manages to express itself in various disorders, together with divergence, referring to how it is that a person with a transdiagnostic process develops a specific type of disorder and not another. These aspects remain to be fully defined until now (Liew et al., 2023).

#### 1.1.1 Stress in university students

University life is a context in which young people face a series of experiences that could affect their well-being (Rossi et al., 2019); possible sources of stress are greater study demands, academic failure, changes in the social support network, and demands for greater protagonism and autonomy, among others (Micin and Bagladi, 2011). This is intensified in middle- and low-income countries such as Chile, where students from lower sociodemographic strata have poorer school preparation, lower financial resources, and availability of support networks with skills to accompany this transition process (Evans-Lacko and Thornicroft, 2019). In Chile, the population entering higher education has increased, including people with greater economic vulnerability, from ethnic groups, residents of rural areas, and/or those who correspond to the first generation of university students in their family (Araneda-Guirriman et al., 2018), all of whom may be more vulnerable to the stress of this context.

A systematic review of risk factors for mental health in university students concluded that the stress experienced by university students interacts – through the stress diathesis model – with other risk factors found in this population, especially cognitive vulnerability: repetitive negative thinking, brooding, negative coping and problem-solving avoidance, perceived burdensomeness, and thwarted belongingness (Sheldon et al., 2021).

One factor that has affected university mental health in this decade has been the COVID-19 pandemic; one study found that three out of four Chilean university students had worsened their mental health during the year 2020 (Mac-Ginty et al., 2021). Multi-country studies also reported high prevalence levels in university students during the pandemic (Ochnik et al., 2021), and a decrease in euthymia, positive mental health, resilience, and well-being has also been observed among university students (see Carrozzino et al., 2021).

### 1.2 Repetitive negative thinking (RNT)

Repetitive negative thinking is a cognitive process characterized by recurrent thoughts and self-focused attention in the face of negative events, whose effects can be adaptive or maladaptive (Cova et al., 2019; Lawrence et al., 2021); the most well-known repetitive thinking is the maladaptive one, usually referred to as RNT, that includes negative rumination, and worry, while on the adaptive side is reflection (Nolen-Hoeksema and Watkins, 2011; Cova et al., 2019; Lawrence et al., 2021). Although repetitive negative thinking is employed as a strategy for self-regulation and modulating own affective experiences, it may be counterproductive, and it has been associated with affective disorders (Ferrer et al., 2018; Spinhoven et al., 2018), eating disorders (Palmieri et al., 2021), increased vulnerability to stress-related disorders in those exposed to childhood adversities (Mansueto et al., 2021), difficulties in emotion regulation (Mansueto et al., 2022, 2024; Palmieri et al., 2023), perfectionism (Kummer et al., 2024; Palmieri et al., 2024) and shyness (Palmieri et al., 2018).

Nolen-Hoeksema (1991) defined depressive rumination as passive and repetitive thinking about one’s own depressive symptoms and their possible causes and consequences; she considered it a tendency or style of thinking stable over time that increases and prolongs depressive symptoms, increasing the probability that they become chronic. Over the years, a more detailed analysis of the scale used to study depressive rumination (Ruminative Response Scale, RRS) made it possible to distinguish two components of rumination: reflection and brooding (Treynor et al., 2003). The latter was conceptualized not only as a response to depressive symptoms but also to negative events or stressors in general (Nolen-Hoeksema et al., 2008). To this day, the distinctive elements of brooding include an orientation towards the past and an evaluative component of the experience in a negative way (Watkins and Roberts, 2020). Conversely, reflection was defined as turning inward to perform cognitive problem-solving to alleviate distress symptoms (Treynor et al., 2003). Subsequently, other authors have defined a construct similar to reflection, adaptive rumination, which is characterized as repetitive thinking focused on understanding the negative process experienced and the search for solutions (Watkins, 2008).

Brooding as a thinking style has been widely studied as a transdiagnostic process, accumulating cross-sectional evidence on its relationship with depressive and anxiety disorders, and suicidal ideation (Tucker et al., 2013; Ferrer et al., 2018). Longitudinal studies in the Chilean population have established that negative rumination influences the development of depressive and anxiety symptoms (Cova et al., 2009; Pimentel and Cova, 2011), and international evidence shows the impact of rumination on the development of suicidal ideation at one-year follow-up (Miranda and Nolen-Hoeksema, 2007).

Reflection or adaptive rumination has less empirical support to date (Cova et al., 2019; Lawrence et al., 2021). One element that has been debated is whether it operates as a protective factor against stress since, although it is associated with fewer symptoms in cross-sectional studies, it is usually related to brooding at follow-ups (Cova et al., 2009, 2019). Some characteristics proposed to consider reflection as adaptive are the attributes of being deliberate, controllable, concrete, and self-distancing, although several of these elements have little research (Cova et al., 2019; Lawrence et al., 2021).

Worry is defined as a chain of thoughts and images focused on the potential negative implications of possible negative events, such as being fired, failing an exam, getting sick, having an accident, or even positive ones, such as a promotion, love relationship, etc. (Papageorgiou, 2006). Initially, it was associated exclusively with generalized anxiety (Newman et al., 2013), but over time, evidence of its action as a transdiagnostic process for other anxiety disorders, depression, pain disorder, eating disorders, and psychosis was gathered (Ehring and Behar, 2020). In Chile, a one-month follow-up study of RNT did not establish the influence of worry on the development of anxious symptoms in university students but of brooding (Pimentel and Cova, 2011).

In the last decade, the field of study of RNT has expanded the focus of determining what type or content of negative repetitive thought-ruminating or worrying is associated with which disorders to know which RNT processes make it maladaptive. Among the attributes proposed for the study are intrusiveness, lack of control, duration, and self-immersive or first-person perspective (Cova et al., 2019; Rosenkranz et al., 2020; Lawrence et al., 2021); elements that are usually assessed with Perseverative Thinking Questionnaire (PTQ). Studies show cross-sectional associations between process RNT and social anxiety and depression in adolescents (Klemanski et al., 2017). It has also been observed that the presence of higher RNT allows distinguishing people with different anxiety disorders and depression and differentiating them from healthy controls (Wahl et al., 2019). Factor analyses of the PTQ in patients with social anxiety found that pre- and post-event RNT clustered into a single RNT factor (Wong et al., 2019), leading the authors to postulate that content toward the future (worry) or toward the past (brooding) is less relevant than process RNT as transdiagnostic elements. A five-year longitudinal investigation of people with anxiety and depressive disorders found that process RNT mediated progression between diagnostics, usually from anxiety to depression (Spinhoven et al., 2019).

### 1.3 RNT and ecological momentary assessment

Traditional RNT studies used cross-sectional designs using questionnaires, which had limitations such as difficulties in establishing causality in relationships and recall biases due to depressive mood, among others (Connolly and Alloy, 2017; Bravo et al., 2019; Hamonniere et al., 2020). Ecological momentary assessment (EMA) overcomes this difficulty; this design involves the repeated sampling of participants’ behaviors and experiences in real-time, in their natural context (Shiffman et al., 2008). In EMA studies, reactive RNT is referred to as an RNT that is assessed moment-to-moment to distinguish it from RNT styles assessed with questionnaires at baseline.

EMA studies have provided prospective evidence with ecological validity on RNT. Connolly and Alloy (2017) developed an EMA study where reactive rumination predicted increased depressive symptoms in the face of stress in a seven-day follow-up study; furthermore, there was an interaction between stress and reactive rumination in predicting increased depressive symptoms. Another study was able to establish the influence of suicide-specific reactive rumination on the completion of a suicide attempt in an 18-day follow-up of patients with suicidal ideation (Rogers et al., 2021). In relation to worry, Newman et al. (2019) conducted an eight-day follow-up study of patients with anxiety and healthy controls, observing that reactive worry is associated with greater anxiety in the following hour. In line with the study of RNT types and process, a follow-up with EMA detected that RNT process, repetition, intrusiveness, duration, and perceived burden influenced the development of anxious-depressive symptoms and stress at 14 days and were more relevant than RNT types (Rosenkranz et al., 2020).

The present study used ecological momentary assessment to evaluate a transdiagnostic model that includes RNT and stress for the one-week prediction of eight groups of psychiatric symptoms. We hypothesized that higher levels of university life stress and reactive repetitive negative thinking would increase symptom development beyond RNT styles and previous-term academic stress. We also expected different effects of moment-to-moment stress and reactive RNT on the different syndromes (divergence).

## 2 Materials and methods

### 2.1 Participants

It had been estimated that a sample of 155 persons was required considering an F-test for multiple linear regression with 10 predictors, with an expected R^2^ of 10% incremental over any previous model, with a significance level of 5% and a power of 80%. In total, 309 students aged 18 to 25 years from various faculties of a Chilean university filled out the baseline questionnaires. Following previous studies in EMA (Rosenkranz et al., 2020), the final sample consisted of 238 participants (77.0%) who met the criteria defined for the analyses: responding to the day 1 baseline assessment, responding to the EMA assessment on at least 3 days, with at least 13 measurements in total. Of these, 121 were female (56.7%) and 117 were male (42.3%), with an average age of 20.9 years (SD = 1.79), 69 (29.0%) had been in mental health treatment in the past year, and 54 (22.7%) had received a diagnosis of mental disorder in the past year.

### 2.2 Measures

#### 2.2.1 Self-report questionnaires

Ruminative Response Scale, RRS (Treynor et al., 2003; Cova et al., 2007). Self-report designed to evaluate the ruminative tendency of people when feeling emotional distress. The scale has two subscales of five items each, reflection, and brooding. In Chile, the version of Cova et al. (2007) is used, in which two items per subscale have been added to improve reliability (Cova et al., 2009). In university students, it has a reliability of 0.69 for reflection and 0.81 for negative rumination (Pimentel and Cova, 2011). In the current study, internal consistency was *α* = 0.80 for the brooding scale and *α* = 0.73 for the reflection scale.

Penn State Worry Questionnaire, PSWQ (Meyer et al., 1990; Sandín et al., 2009). A 16-item self-report measure attempts to measure the frequency and intensity of worry in general and the subject’s difficulty in controlling it. It has a Spanish version and an Argentine adaptation with good psychometric properties (Rodríguez and Vetere, 2006; Sandín et al., 2009). Reliability in Chilean university students is 0.94 (Pimentel and Cova, 2011). In the current study, internal consistency was *α* = 0.96.

Symptom Check List, SCL-90-R (Derogatis, 1975; Gempp Fuentealba and Avendaño Bravo, 2008): A 90-item self-administered multidimensional questionnaire that includes scales for somatization, obsessive-compulsive, interpersonal sensitivity, depression, anxiety, phobic anxiety, hostility, paranoid ideation, and psychoticism. It is considered a valid instrument for assessing various psychiatric disorders (Lignier et al., 2024). It has been translated into Spanish, and its use in Chile has shown adequate psychometric properties in university students, with internal consistency ranging from 0.78 to 0.90 in the different subscales (Gempp Fuentealba and Avendaño Bravo, 2008). This study’s internal consistencies ranged from *α* = 0.76 for hostility to 0.93 for somatization.

SISCO-II for Academic Stress (Barraza, 2007; Castillo-Navarrete et al., 2020): The version used is that of Castillo-Navarrete et al. (2020), with 33 items, eight of which identify the frequency of academic stressor stimuli. This study used stressors subscale to evaluate the academic stress of the current term. In Chilean university students, an α 0.77 was determined (Castillo-Navarrete et al., 2020). In the present study, the α was 0.80.

Sociodemographic Form this form includes categorical items for the variables sex, age, semester of current degree, housing: family/other relatives or friends/rents, family income level, work (YES/NO), and mental health diagnosis and treatment in the last year.

#### 2.2.2 Ecological momentary assessment (EMA)

For six consecutive days (days two to eight), participants received five notifications per day, every 3 h, over a 12-h time-window starting at a time selected by them (between 7 and 10 AM for the start). After receiving a notification, participants had two and a half hours to answer 18 questions, starting with “From the last evaluation until now…” about different academic and non-academic stressors and the content and process of RNT (see Table 1). Following previous authors (Rosenkranz et al., 2020), items were selected from RNT questionnaires and adapted to EMA. For RNT content: RRS for negative rumination and reflection, and PSWQ for worry; for RNT process: Perseverative Thinking Questionnaire, PQT (Ehring et al., 2011; Valencia, 2020) for controllability/intrusiveness, and a duration item taken from Rosenkranz et al. (2020). Participants were reminded 30 min before the period ended if they had not answered the questions up to this point. The dimensions of academic stress and non-academic stress were determined through exploratory factor analysis with good indices of fit.

**Table 1:** EMA – items assessing stress and repetitive negative thinking.

Dimension	Items	Scale	Reliability^a^
Academic stress	Have you felt stressed about completing your academic workload? Have you done poorly on a test, exam, or assignment?	Not at all stressed = 0 to very stressed = 4	Punctual: ICC =0.61 Daily: ICC =0.89
Non-academic stress	A problem in your relationships (family, couple, friends)	Not at all stressed = 0 to very stressed = 4	Punctual: ICC = 0.69 Daily: ICC = 0.92
	An embarrassing social situation for you	Not at all stressed = 0 to very stressed = 4	
	An economic problem (not being able to afford some necessary expenses, receive collections, etc.)	Not at all stressed = 0 to very stressed = 4	
	A health problem (illness, accident, etc.)	Not at all stressed = 0 to very stressed = 4	
	A situation where I felt assaulted, harassed, or that my safety was at risk.	Not at all stressed = 0 to very stressed = 4	
RNT-content			
Brooding	I’ve thought, “Why cannot I handle things better?” I’ve been thinking over and over again about the bad things that have happened to me.	Not at all stressed = 0 to very stressed = 4	Punctual: ICC = 0.69 Daily: ICC = 0.89
Worry	My worries overwhelm me. Even if I cannot do anything to change it, I still worry about it. I thought about all the bad things that could happen. If something bad happened to me, I cannot help but think it’s going to get worse.	Not at all stressed = 0 to very stressed = 4	Punctual: ICC =0.74 Daily: ICC =0.94
Reflection	I isolated myself and thought about why I felt this way. I’m trying to figure out why I feel that way.	Not at all stressed = 0 to very stressed = 4	Punctual: ICC = 0.61 Daily: ICC =0.90
RNT-process			
Duration	How long have you been thinking about your difficulties?	Not at all stressed = 0 to very stressed = 4	Daily: ICC =0.89 Total: ICC =0.99
Intrusiveness	The thoughts keep going through my mind again and again. Thoughts come to my mind without me wanting them to. I get stuck in certain problems and cannot move on. Very vivid images of my problems came to me (as if they were happening)	Not at all stressed = 0 to very stressed = 4	Punctual: ICC =0.75 Daily: ICC =0.95

^a^Reliability of the point and daily measurement obtained by the method of Cranford et al. (2006).

### 2.3 Procedure

The study was approved by the Scientific Ethical Committee of the university where it was performed (Certificate number CB1123-2022) and is in accordance with the Declaration of Helsinki. Students were invited to participate on campus via posters and online announcements on social media. In-person, after signing the informed consent protocol, a brief admissibility test was applied on a web page, excluding cases with suicidal risk or psychotic symptomatology. Excluded persons were given feedback and a pamphlet with ways to seek help. Accepted participants were introduced to the EMA app (OURMIND developed at Concepcion University for research use), which they had installed on their smartphone (iOS or Android). Participants received the equivalent of US$24 for their participation upon completing 80% of the assessments.

On day one, participants responded to the SCL-90R, RRS, PSWQ, SISCO II, and a sociodemographic form. The EMA period started on day one, lasted 6 days (days two to seven), and consisted of five daily assessments. Each time, the students answered items about academic and non-academic stress, reactive RNT content, and process. The SCL-90R questionnaire was reevaluated on day eight.

### 2.4 Data analysis

The application data were exported to Excel format and statistical analyses were conducted in R, version 4.3. In this study, missing data were addressed using multiple imputations by Fully Conditional Specification (FCS). This approach is particularly suitable for our dataset, which exhibits a complex pattern of missingness (Liu and De, 2015). The imputation process was carried out through 20 iterations in each of the 20 imputed datasets, a number determined to be sufficient for convergence based on diagnostic checks. Each variable with missing data was imputed conditionally on all other variables in the model, using an appropriate model for each variable type (e.g., logistic regression for binary variables and linear regression for continuous variables). After the imputation process, analyses were performed on each of the 20 datasets separately, and the results were then combined using standard rules for multiple imputation inference. SCL-90 R Anxiety and Phobic Anxiety subscale scores were summed as in a common anxiety scale (Saldivia et al., 2023).

Descriptive analyses of the self-report questionnaires and EMA variables assessed were performed. The reliability of the questionnaire measures – symptom measurements with SCL-90R and RNT styles – was calculated using Cronbach’s alpha. An exploratory factor analysis was used to define academic and non-academic stress dimensions. The number of factors was estimated using Horn’s parallel analysis, using the least squares extraction method and oblimin rotation. To estimate the reliability of the EMA measures, EMA-stress and EMA-RNT, the method proposed by Cranford et al. (2006), was used through intraclass correlation.

To test the predictive power of the study variables in the development of SCL-90R symptoms, seven hierarchical stepwise linear regression models were used, organized according to a temporal criterion defined by the sequence of data collection and theoretical criteria. The first four models respond to variables obtained from the first measurement (baseline, day one): Model 1 considers only sociodemographic; Model 2 adds the baseline psychiatric symptoms (SCL-90R); Model 3 includes the RNT style: brooding, worry and reflection (RRS and PSQW); Model 4 adds the term academic stress (SISCO-II). Models 5 to 7 include the EMA measurements (days two to seven). Model 5 adds EMA- academic and non-academic stress. Model 6 includes the EMA-RNT process, which would arise from the EMA-stress. Finally, EMA-RNT content is added in model 7.

To determine the predictive power of each model, R^2^ values were estimated; to determine the differences in predictive value between each model, Rubin’s D1, analogous to an Anova for missing data (*F* value), was used as a test. The relative fit of the models to the data was tested using AIC and BIC to determine the best model combining parsimony with the best predictive power, where lower AIC and BIC values reflect a better fit. Since the comparison between models using AIC and BIC must be performed on the same database, it is not possible to choose the AIC or BIC with the lowest average among the imputed bases; therefore, the strategy used was to identify, for each imputed base, which was the model with the lowest AIC and BIC, and in cases where both indicators corresponded to different models, the model with the lowest AIC in most of the bases was chosen (Vrieze, 2012).

## 3 Results

### 3.1 Descriptive data

Table 2 shows the descriptive statistics of the study scales with the grand mean of each scale. The grand mean of syndromic groups in SCL-90R ranged from 0.58 for psychoticism to 1.58 for obsessions, being equivalent to other Chilean samples of university students (Gempp). In the case of repetitive thinking styles, grand mean were 2.18 for rumination to 3.00 for worry. Although the results for rumination and reflection are similar to previous samples, the grand mean for worry is higher in this sample (Pimentel and Cova, 2011). Academic stress during the semester obtained a grand mean of 2.97, equivalent to a study in a similar sample (Guzmán-Castillo et al., 2022).

**Table 2:** Descriptive statistics of repetitive thinking style and SCL-90R in initial evaluation and follow-up.

	Day 1						Day 8					
	M _item_	SD	Min	Max	skew	kurtosis	M _item_	SD	Min	Max	skew	kurtosis
Brooding	2.18	0.61	1.00	3.71	0.31	−0.53						
Reflection	2.34	0.56	1.14	4.00	0.30	−0.44						
Worry	3.00	0.95	1.13	5.00	0.04	−0.92						
Term academic stress	2.97	0.68	1.38	5.00	−0.02	0.04	2.64	0.84	1	5	0.19	−0.49
SCL-90R												
Depression	1.42	0.80	0.00	3.33	0.31	−0.88	1.36	0.77	0	3.23	0.30	−0.66
Anxiety	1.08	0.85	0.00	4.00	1.02	0.67	1.03	0.89	0	4.00	1.05	0.47
Hostility	0.63	0.57	0.00	3.17	1.56	3.11	0.62	0.58	0	3.50	1.81	4.33
Paranoia	0.78	0.72	0.00	3.17	1.01	0.52	0.64	0.65	0	3.33	1.14	1.31
Obsessions	1.58	0.88	0.00	3.80	0.15	−0.72	1.39	0.96	0	3.80	0.38	−0.73
Somatization	0.92	0.84	0.00	3.50	1.28	0.91	0.84	0.87	0	4.00	1.58	2.27
Interpersonal Sensitivity	1.07	0.79	0.00	3.44	0.65	−0.36	0.90	0.81	0	3.44	0.84	−0.06
Psychoticism	0.58	0.49	0.00	2.20	0.89	0.10	0.50	0.52	0	2.60	1.24	1.19

Maximum possible range: Brooding, reflection, worry = 1–5. Term stress: 1–5. SCL-90R scales: 0–4.

Table 3 shows descriptive data of the EMA items with grand means of the EMA-stress and EMA-RNT process and content scales. It is not possible to compare the obtained data with any existing studies. Although, it is possible to observe that the EMA-stress means are less than half of the possible range. In addition, it stands out that the average EMA academic stress experienced by students is higher than EMA non-academic stress. In the case of EMA-RNT content, the frequencies of brooding, reflection and worry content are low compared to the maximum possible, with reflection being the least frequent in the face of stress. In relation to the EMA-RNT process, duration, and intrusion are low in relation to the maximum possible, being the average of EMA-RNT process: duration, higher than that of EMA-RNT process: intrusion.

**Table 3:** Descriptive data of EMA-stress and EMA-RNT.

	M _item_	SD	Min	Max	skew	kurtosis
EMA–academic stress	1.27	1.40	0	4	0.72	−0.84
EMA-non academic stress	0.63	1.05	0	4	1.72	2.14
EMA-RNT content: brooding	0.72	1.09	0	4	1.51	1.43
EMA-RNT content: reflection	0.48	0.92	0	4	2.08	3.84
EMA-RNT content: worry	0.63	1.06	0	4	1.71	2.05
EMA-RNT process: duration	1.03	1.16	0	4	0.95	−0.03
EMA-RNT process: intrusiveness	0.42	0.90	0	4	2.38	5.24

Maximum possible range = 0–4.

### 3.2 SCL-90R symptom prediction based on study variables

According to our data analysis strategy, seven hierarchical stepwise linear regression models were utilized to assess the power of the study’s variables to predict changes in SCL-90R symptoms. These models considered: Model 1: sociodemographic information; Model 2: added initial symptom severity (day one); Model 3: added RNT styles (brooding, reflection, and worry); Model 4: added term academic stress; Model 5: added EMA–academic and non-academic stress; Model 6: added EMA–RNT process (duration and intrusiveness); and EMA-RNT content (reactive brooding, reflection, and worry). Table 4 presents the fit data of the hierarchical regression models for depressive, anxiety, and somatization symptoms, considering R^2^, change in R^2^, AIC, and BIC.

**Table 4:** R^2^, Delta R^2^, AIC y BIC of the hierarchical linear regression models for depression, anxiety, and somatization symptoms.

	Depression						Anxiety							Somatization						
Model	R^2^	F	P	Delta R^2^	AIC	BIC		R^2^	F	P	Delta R^2^	AIC	BIC		R^2^	F	P	Delta R^2^	AIC	BIC
**1**: Sociodemographic.	0.106	–	–	–	540.8	572.0	**1**	0.112	–	–	–	605.5	636.8	**1**	0.145	–	–	–	590.8	622.1
**2**: Added SCL-90R day one.	0.532	176.3	0,001	0.426	385.8	420.6	**2**	0.604	204.2	0,001	0.491	415.4	450.1	**2**	0.591	184.0	0,001	0.446	417.3	452.0
**3**: Added RNT style	0.541	1.1	0.352	0.008	388.3	433.4	**3**	0.615	1.6	0.188	0.010	414.9	460.0	**3**	0.595	0.6	0.638	0.004	420.9	466.1
**4**: Added term academic stress	0.564	10.2	0.002	0.022	377.7	426.3	**4**	0.619	1.9	0.168	0.005	413.8	462.4	**4**	0.596	0.1	0.890	0.001	422.7	471.4
**5**: Added EMA - stress	0.611	10.6	0,001	0.046	356.2	411.7	**5**	0.666	12.1	0,001	0.046	386.6	442.1	**5**	0.621	6.3	0.002	0.026	411.2	466.8
**6**: Added EMA-RNT process	0.621	2.6	0.078	0.010	353.4	415.9	**6**	0.669	0.8	0.458	0.003	388.3	450.8	**6**	0.631	2.6	0.073	0.010	408.6	471.1
**7**: Added EMA-RNT content	0.623	0.1	0.979	0.002	358.3	431.2	**7**	0.673	0.5	0.704	0.004	391.7	464.6	**7**	0.636	0.6	0.603	0.005	411.6	484.6

In the case of the prediction of depressive symptoms, there are significant differences between models 1 and 2 (*p* = 0.001), model 3 and 4 (*p* = 0.002) and between 4 and 5 (*p* = 0.001), which would indicate the differential contribution of baseline symptoms, semester stress and week stress, respectively. When analyzing the lowest AIC in the different imputed bases, we can observe that the best model is 6 (Table 4), which adds to the baseline predictors, and the EMA-stress, the EMA-RNT process (R^2^ = 0.62). Table 5 presents the coefficients of multiple regression model 6 for the prediction of depressive symptoms. Significant in this model are the regression coefficients: sociodemographic – housing = other relatives or friends, *b* = 0.19, *p* = 0.039, baseline depressive symptoms, *b* = 0.49, *p* = 0.001, and the coefficient of RNT-process: duration, *b* = 0.24, *p* = 0.019.

**Table 5:** Selected hierarchical linear regression model for depression symptoms on day 8.

Step		B	p
0	Intercept	−0.13	0.820
1	Sex = male	−0.04	0.626
	Age	−0.02	0.572
	Year in university program	0.01	0.728
	**Housing = other relatives or friends**	**0.19**	0.039*
	Housing = dorms or rent	0.04	0.624
	Income level	0.03	0.066
	Work: yes	−0.05	0.692
2	**SCL-90R Day 1: depression**	**0.49**	0.001***
3	Brooding (RRS)	−0.02	0.735
	Reflection (RRS)	0.07	0.298
	Worry (PSQW)	−0.03	0.561
4	Term academic stress	0.12	0.107
5	EMA-academic stress	−0.01	0.843
	EMA-non-academic stress	0.03	0.856
6	EMA-RNT process: intrusiveness	0.06	0.637
	**EMA-RNT process: duration**	**0.24**	0.019*

Significant coefficients denoted in bold. ^***^p < 0.001, ^**^p < 0.01, ^*^p < 0.05.

For the prediction of anxiety symptoms (Table 4), significant differences are observed between models 1 and 2 (*p* = 0.001) and between 4 and 5 (*p* = 0.002), which would indicate the differential contribution of incorporating baseline symptoms and EMA-stress, respectively. When analyzing the lowest AIC in the different imputed bases, we can observe that the best model is 5, which obtains the lowest BIC (R^2^ = 0.67). This model adds EMA-stress to the baseline predictors of anxiety symptoms, RNT style and term academic stress. Of the regression coefficients of model 5 (Table 6), the coefficients of anxiety symptoms on day 1 are significant (*b* = 0.61, *p* = 0.001), as well as the coefficient of EMA non-academic stress (*b* = 0.29, *p* = 0.001).

**Table 6:** Selected hierarchical linear regression models for anxiety symptoms on day 8.

Step		B	p
0	Intercept	−0.11	0.877
1	Sex = male	−0.08	0.312
	Age	−0.02	0.441
	Year in university program	0.02	0.709
	Housing = other relatives or friends	0.05	0.610
	Housing = dorms or rent	−0.01	0.918
	Income level	0.02	0.316
	Work: yes	−0.02	0.869
2	**SCL-90R Day 1: anxiety**	**0.61**	0.001***
3	Brooding (RRS)	0.03	0.672
	Reflection (RRS)	0.06	0.443
	Worry (PSQW)	0.03	0.649
4	Term academic stress	−0.03	0.655
5	EMA-academic stress	0.08	0.206
	**EMA-non-academic stress**	**0.29**	0.001***

Significant coefficients denoted in bold. ^***^p < 0.001, ^**^p < 0.01, ^*^p < 0.05.

When analyzing the models predicting somatization symptoms (Table 4), we can observe significant differences between models 1 and 2 (*p* = 0.001) and between 4 and 5 (*p* = 0.002), which would indicate a differential contribution of including baseline symptoms and EMA-stress, respectively. When analyzing the lowest AIC in the different imputed bases, we can observe that the best model is 6, where the EMA-RNT process is added to the four groups of predictors of baseline and EMA-stress, with an R^2^ = 0.63. Reviewing the regression coefficients of model 6 (Table 7), the coefficient of somatization on Day 1 (*b* = 0.691, *p* = 0.001), and the coefficient of EMA-RNT: duration (*b* = 0.245, *p* < 0.001), are significant.

**Table 7:** Selected hierarchical linear regression models for somatization symptoms on day 8.

Step		B	p
0	Intercept	0.66	0.325
1	Sex = male	−0.09	0.275
	Age	−0.04	0.214
	Year in university program	0.01	0.958
	Housing = other relatives or friends	0.02	0.836
	Housing = dorms or rent	−0.07	0.419
	Income level	−0.01	0.959
	Work: yes	−0.15	0.304
**2**	**SCL-90R Day 1: somatization**	**0.69**	0,001***
3	Brooding (RRS)	0.02	0.822
	Reflection (RRS)	0.02	0.751
	Worry (PSQW)	0.01	0.857
4	Term academic stress	−0.11	0.123
5	EMA-academic stress	0.01	0.920
	EMA-non-academic stress	−0.13	0.478
6	EMA-RNT process: intrusiveness	0.16	0.299
	**EMA-RNT process: duration**	0.25	0.027**

Significant coefficients denoted in bold. ^***^p < 0.001, ^**^p < 0.01, ^*^p < 0.05.

Table 8 contains the fit data of the hierarchical linear regression models of paranoia, psychoticism, and obsession symptoms, including R^2^, change in R^2^, AIC and BIC.

**Table 8:** R^2^, Delta R^2^, AIC y BIC of the hierarchical linear regression models for paranoia, psychoticism, and obsessions symptoms EN DÍA 8.

	Paranoia							Psicoticismo							Obsesiones					
Model	R^2^	F	P	Delta R^2^	AIC	BIC		R^2^	F	P	Delta R^2^	AIC	BIC		R^2^	F	P	Delta R^2^	AIC	BIC
**1**: Sociodemographic.	0.068	–	–	–	475.9	507.1	**1**	0.088	–	–	–	366.2	397.4	**1**	0.092	–	–	–	645.8	677.0
**2**: Added SCL-90R day one.	0.502	149.4	**0.001**	0.434	328.9	363.6	**2**	0.489	156.1	**0.001**	0.401	230.0	264.7	**2**	0.498	175.2	**0.001**	0.405	507.1	541.8
**3**: Added RNT style	0.514	1.478	0.222	0.012	329.1	374.2	**3**	0.499	1.196	0.312	0.009	231.4	276.5	**3**	0.509	1.424	0.237	0.012	507.5	552.7
**4**: Added Term academic stress	0.516	0.877	0.350	0.003	329.8	378.4	**4**	0.506	2.388	0.124	0.006	230.4	279.0	**4**	0.516	2.113	0.149	0.006	506.5	555.1
**5**: Added EMA - stress	0.529	2.412	0.093	0.013	327.1	382.6	**5**	0.533	5.183	**0.006**	0.027	221.0	276.5	**5**	0.568	10.61	**0.001**	0.053	482.8	538.3
**6**: Added EMA-RNT process	0.548	3.59	**0.030**	0.018	321.8	384.3	**6**	0.570	8.216	**0.001**	0.037	205.1	267.6	**6**	0.582	2.928	0.056	0.014	479.1	541.6
**7**: Added EMA-RNT content	0.561	1.492	0.218	0.013	321.0	393.9	**7**	0.585	1.868	0.137	0.015	202.8	275.7	**7**	0.593	1.358	0.257	0.010	479.1	552.0

Significant coefficients denoted in bold.

When comparing the seven hierarchical models for predicting paranoia symptoms (Table 8), significant differences exist between models 1 and 2 (*p* = 0.001) and between 5 and 6 (*p* = 0.003), which would indicate the differential contribution of adding the baseline symptoms and the EMA-RNT process, respectively. When analyzing the lowest AIC in the different imputed bases, we can observe that the best model is 6, also with the lowest BIC; this model adds to the baseline predictors and the EMA-stress, the EMA-RNT process (R^2^ = 0.55). Regression coefficients for paranoia symptoms in model 6 are presented in Table 9. The coefficient of paranoia symptoms at baseline (*b* = 0.54, *p* = 0.001) and EMA-RNT process: duration (*b* = 0.25, *p* = 0.001) are significant.

**Table 9:** Selected hierarchical linear regression models for paranoia symptoms.

Step		B	P
	Intercept	0.19	0.739
1	Sex = male	−0.02	0.741
	Age	−0.02	0.379
	Year in university program	0.03	0.439
	Housing = other relatives or friends	0.06	0.525
	Housing = dorms or rent	−0.09	0.206
	Income level	0.01	0.807
	Work: yes	0.01	0.949
2	**SCL-90R Day 1: paranoia**	**0.54**	0.001***
3	Brooding (RRS)	0.05	0.453
	Reflection (RRS)	0.01	0.907
	Worry (PSQW)	0.02	0.622
4	Term academic stress	0.03	0.624
5	EMA-academic stress	−0.12	0.063
	EMA-non-academic stress	0.01	0.994
6	EMA-RNT process: intrusiveness	−0.04	0.743
	**EMA-RNT process: duration**	**0.25**	0.007**

Significant coefficients denoted in bold. ^***^p < 0.001, ^**^p < 0.01, ^*^p < 0.05.

In Table 8, there is a significant difference between models 1 and 2 (*p* = 0.001), between 4 and 5 (*p* = 0.006), and between 5 and 6 (*p* = 0.001) in predicting psychotic symptoms, which would indicate the differential contribution of adding the baseline symptoms, the EMA-stress, and the EMA-RNT process, respectively. Observing the AIC indices in the different imputed bases, we can see that the model with the lowest AIC is 7, which adds the EMA-RNT content to the predictors to the baseline predictors, EMA-stress and EMA-RNT process, with an R^2^ = 0.59. The regression coefficients of model 7 are in Table 10, where the coefficient of psychotic symptoms on day 1 (*b* = 0.63, *p* = 0.001), and the coefficient of EMA-RNT duration (*b* = 0.19, *p* = 0.024), are significant.

**Table 10:** Selected hierarchical linear regression models for psychoticism symptoms.

Step		B	P
	Intercept	0.03	0.944
1	Sex = male	−0.04	0.424
	Age	−0.01	0.508
	Year in university program	0.01	0.755
	Housing = other relatives or friends	0.10	0.138
	Housing = dorms or rent	−0.04	0.518
	Income level	−0.01	0.503
	Work: yes	−0.08	0.399
2	**SCL-90R Day 1: psychoticism**	**0.63**	0.001***
3	Brooding (RRS)	0.08	0.084
	Reflection (RRS)	0.01	0.785
	Worry (PSQW)	−0.06	0.095
4	Term academic stress	0.03	0.576
5	EMA-academic stress	−0.07	0.149
	EMA-non-academic stress	−0.98	0.282
6	EMA-RNT process: intrusiveness	0.17	0.1693
	**EMA-RNT process: duration**	**0.19**	0.024*
7	EMA_RNT content: reflection	0.09	0.152
	EMA-RNT content: brooding	0.24	0.132
	EMA-RNT content: worry	0.57	0.466

Significant coefficients denoted in bold. ^***^p < 0.001, ^**^p < 0.01, ^*^p < 0.05.

When reviewing the fit data of the hierarchical regression models for the prediction of obsessive symptomatology (Table 8), we can observe significant differences between models 1 and 2 (*p* = 0.001) and between 4 and 5 (*p* = 0.001), which would indicate a differential contribution of including baseline symptoms and EMA – stress, respectively. When analyzing the lowest AIC in the different imputed bases, we can observe that the best model is 6, also with the lowest BIC. In this model, the EMA-RNT content is added to the baseline and EMA-stress predictors, with an R^2^ = 0.58. Reviewing the regression coefficients of model 6 (Table 11), we see that the coefficient of obsessive symptoms on day 1 (*b* = 0.56, *p* = 0.001), and the coefficient of EMA-RNT duration (*b* = 0.30, *p* = 0.023), are significant.

**Table 11:** Selected hierarchical linear regression model for obsessions symptoms.

Step		B	P
	Intercept	−0.18	0.819
1	Sex = male	−0.02	0.842
	Age	−0.03	0.497
	Year in university program	0.04	0.477
	Housing = other relatives or friends	0.15	0.215
	Housing = dorms or rent	−0.05	0.656
	Income level	0.02	0.237
	Work: yes	−0.26	0.129
2	**SCL-90R Day 1: obsessions**	**0.56**	0.001***
3	Brooding (RRS)	0.10	0.217
	Reflection (RRS)	−0.03	0.766
	Worry (PSQW)	−0.02	0.819
4	Term academic stress	−0.01	0.932
5	EMA-academic stress	0.11	0.185
	EMA-non-academic stress	−0.19	0.359
6	EMA-RNT process: intrusiveness	0.21	0.242
	**EMA-RNT process: duration**	**0.30**	0.022*

Significant coefficients denoted in bold. ^***^p < 0.001, ^**^p < 0.01, ^*^p < 0.05.

Finally, Table 12 contains the model fit data of hostility and interpersonal sensitivity syndromes, including R^2^, change in R^2^, AIC, and BIC.

**Table 12:** R^2^, Dif. R^2^, AIC y BIC of the hierarchical linear regression models for hostility and interpersonal sensitivity symptoms.

	Hostility							Interpersonal sensitivity					
Model	R^2^	F	P	Delta R^2^	AIC	BIC		R^2^	F	P	Delta R^2^	AIC	BIC
**1**: Sociodemographic.	0.103				436.7	507.1	**1**	0.094				571.6	602.9
**2**: Added SCL-90R day 1.	0.388	75.75	**0.001**	0.285	351.2	363.6	**2**	0.575	225.7	**0.001**	0.481	393.5	428.2
**3**: Added RNT style	0.424	3.845	**0.010**	0.036	353.3	374.2	**3**	0.581	0.861	0.462	0.006	396.0	441.1
**4**: Added term academic stress	0.428	1.179	0.280	0.004	357.0	378.4	**4**	0.585	1.495	0.224	0.004	395.7	444.3
**5**: Added EMA - stress	0.477	7.854	**0.001**	0.049	346.5	382.6	**5**	0.598	2.646	0.074	0.012	392.5	448.0
**6**: Added EMA-RNT process	0.482	0.777	0.461	0.005	355.1	384.3	**6**	0.611	2.994	0.052	0.013	388.6	451.1
**7**: Added EMA-RNT content	0.503	1.951	0.123	0.021	361.8	393.9	**7**	0.617	0.754	0.521	0.007	390.5	463.4

Significant coefficients denoted in bold.

In the case of hostility symptoms (Table 12), there are significant differences in the predictive capacity between models 1 and 2 (*p* = 0.001), between 2 and 3 (*p* = 0.010), and between 4 and 5 (*p* = 0.001), which would indicate the differential contribution of including the baseline symptoms, the RNT styles, and the EMA-stress. When analyzing the lowest AIC in the different imputed bases, we can observe that the best model is 5, which adds the EMA-stress to the baseline predictors (R^2^ = 0.48). When analyzing the regression coefficients of model 5 (Table 13), the coefficient of hostility symptoms on day 1 (*b* = 0.43, *p* = 0.001), and the coefficient of EMA-non-academic stress (*b* = 0.22, *p* = 0.001) are significant.

**Table 13:** Selected hierarchical linear regression model for hostility symptoms.

Step		B	P
	Intercept	0.07	0.892
1	Sex = male	0.02	0.707
	Age	−0.02	0.407
	Year in university program	−0.02	0.538
	Housing = other relatives or friends	0.18	0.028
	Housing = dorms or rent	−0.04	0.572
	Income level	0.02	0.052
	Work: yes	−0.15	0.171
2	**SCL-90R Day 1: hostility**	**0.43**	0.001***
3	Brooding (RRS)	0.05	0.309
	Reflection (RRS)	−0.02	0.798
	Worry (PSQW)	0.05	0.257
4	Term academic stress	−0.01	0.823
5	EMA-academic stress	0.01	0.863
	**EMA-non-academic stress**	**0.22**	0.001***

Significant coefficients denoted in bold. ^***^p < 0.001, ^**^p < 0.01, ^*^p < 0.05.

Observing the models for the prediction of interpersonal sensitivity symptoms (Table 12), significant differences are observed between models 1 and 2 (*p* = 0.001), which would indicate the differential contribution of incorporating the baseline symptoms. When analyzing the lowest AIC in the different imputed bases, we can observe that the best model is model 6, which adds the EMA-RNT process to the predictors to the baseline predictors, and EMA-stress, with an R^2^ = 0.61. Table 14 presents the coefficients of regression model 6 for interpersonal sensitivity symptoms, where the coefficient of symptoms at baseline (*b* = 0.70, *p* = 0.001) and the coefficient of EMA-RNT duration (*b* = 0.28, *p* = 0.012) are significant.

**Table 14:** Selected hierarchical linear regression model for interpersonal sensitivity symptoms.

Step		B	P
	Intercept	0.03	0.965
1	Sex = male	−0.02	0.823
	Age	−0.03	0.416
	Year in university program	0.02	0.662
	Housing = other relatives or friends	0.14	0.199
	Housing = dorms or rent	−0.01	0.956
	Income level	0.01	0.883
	Work: yes	−0.05	0.741
2	**SCL-90R Day 1: interpersonal sensitivity**	**0.71**	0.001^***^
3	Brooding (RRS)	0.03	0.689
	Reflection (RRS)	0.07	0.344
	Worry (PSQW)	−0.03	0.609
4	Term academic stress	0.03	0.709
5	EMA-academic stress	−0.02	0.793
	EMA-non-academic stress	−0.14	0.428
6	EMA-RNT process: intrusiveness	0.05	0.743
	**EMA-RNT process: duration**	**0.28**	0.012*

Significant coefficients denoted in bold. ^***^p < 0.001, ^**^p < 0.01, ^*^p < 0.05.

## 4 Discussion

This study aimed to determine the effect of stress and RNT in predicting the development of symptoms of various mental health syndromes in university students over 7 days. Multiple assessment variables were used, including baseline symptomatology, RNT styles – brooding, reflection, and worry – and academic stress accumulated during the school period. These traditional predictors were complemented by monitoring for 6 days, through Ecological Momentary Assessment, the levels of academic and non-academic stress (EMA-stress) that the students faced in their university life, together with the RNT activated by stress (EMA-RNT). A particular characteristic of the study is that it evaluated not only the contents of reactive RNT (brooding, reflection, and worry) but also elements of the RNT process, such as its duration and intrusiveness levels (Rosenkranz et al., 2020). Another distinctive quality is that it provides evidence in the Spanish-speaking population of transdiagnostic processes usually studied in English speakers (Hall et al., 2021).

The main results indicate that, after considering baseline symptoms, including RNT styles to the model only increased the predictive power for developing hostility symptoms. Meanwhile, adding EMA-academic and non-academic stress increased the models’ predictive power for the development of depression, anxiety, somatization, obsessive-compulsive, hostility, and psychotic symptoms. On the other hand, adding the EMA-RNT process characteristics increased the prediction of paranoia and psychotic symptoms.

The importance of the predictors EMA-stress and EMA-RNT is reinforced when comparing the fit data of the different hierarchical regression models. In these analyses, the model with the best fit for the development of anxiety and hostility symptoms was model 5, which added the stress of the week to the baseline predictors. For the depression, somatization, obsessive-compulsive, paranoia, and interpersonal sensitivity syndromes, the models with the best fit were model 6, which added to the baseline predictors and the stress of the last week, the EMA-RNT process of the last week. In the case of psychotic symptoms, the model that suggested a better fit was model 7, which adds the EMA-RNT content to the previous sets of variables.

Finally, when assessing the importance of individual variables in the best-fit regression models – after accounting for baseline symptoms – RNT duration (EMA-RNT process: duration) predicted symptom progression in six of eight symptom clusters, while EMA non-academic stress predicted the remaining two.

We would like to elaborate on the findings related to daily stress, reactive repetitive negative thinking (RNT), and the characteristics of RNT, particularly its duration. Regarding daily stress, the EMA methodology used in the study captured stress as an individual perception, including items to evaluate academic and non-academic stressors– social, economic, health, or risk to personal safety – that young people experienced in their daily lives. University students must face various sources of stress, such as greater study demands and independence than in high school, academic failure, changes in the social support network, family and social problems, role conflicts, and greater demands for protagonism and autonomy. These stresses also include financial stress, personal expectations, a more active social life, alcohol use, and substance abuse. Other academic stressors include exams, homework overload, overlapping deadlines, late study hours, as well as the personality and character of the teacher (Micin and Bagladi, 2011; Pitt et al., 2018; Jurado-Botina et al., 2021; Gardani et al., 2022). Stress has previously been defined as a risk factor for the development of emotional disorders in this group (Sheldon et al., 2021). This study established that the stress experienced by university students increases the levels of symptoms of most of the syndromes studied, except paranoia and interpersonal sensitivity. Also, it was possible to detect that non-academic stress affects young people’s mental health during the academic period in the same or greater way than academic stress. The relevance of non-academic stress in contributing to mental health disorders among university students has been previously identified in retrospective studies by the World Health Organization World Mental Health International College Student Initiative (Karyotaki et al., 2020). However, this research provides new prospective evidence, highlighting the ongoing impact of non-academic stressors on student mental health.

Repetitive negative thinking was the other significant predictor of mental health problems in university students. This psychological process influenced the variation of seven of the eight groups of symptoms, clarifying its role as a transdiagnostic process for somatization, obsessive-compulsive, interpersonal sensitivity, depression, paranoid ideation, psychoticism, and hostility. Initial RNT styles were only relevant to the development of hostility symptoms, and reactive RNT for the remaining six. RNT, initially proposed as rumination by Nolen-Hoeksema (1991), is one of the transdiagnostic psychological processes with the most significant evidence (Nolen-Hoeksema and Watkins, 2011; Cova et al., 2019; Ashely, 2020; Palmieri et al., 2021; Miethe et al., 2022). Our results confirm previous findings on RNT, in this case, reactive RNT, as a transdiagnostic process of emotional disorders (Nolen-Hoeksema and Watkins, 2011; Luca, 2019; Wahl et al., 2019; Ehring and Behar, 2020; Hijne et al., 2020) and expand the growing evidence on its implications for paranoid ideation and psychoticism (Zagaria et al., 2023, for example). This is one of the first studies to determine that RNT is associated with increased interpersonal sensitivity and hostility symptoms. In this area, a study with patients with PTSD observed a relationship between rumination and subsequent hostility (Mathes et al., 2020), and a recent study with a cross-sectional design reported an association between RNT and interpersonal sensitivity (Neshat et al., 2024).

The duration of reactive repetitive negative thinking (RNT) as an important predictor of symptomatology in university students is a finding to comment on. As Nolen Hoeksema noted from the beginning, ruminative or repetitive thoughts maintain or increase symptoms because, among other things, they impede active coping and problem-solving (Nolen-Hoeksema and Watkins, 2011; Cova et al., 2019). The obtained results support this hypothesis because the duration of time spent thinking about problems in stressful situations emerged in this study as the most consistent predictor, rather than the content of those thoughts or their intrusiveness. Persistent RNT can be particularly detrimental for students as it can impede their ability to study effectively. The more time they spend on RNT, the less time they have to concentrate on their studies, resulting in increased academic difficulties. Considering these forms of coping as ingrained habits, strategies for modifying this particular habit should be carefully incorporated into programs to cope with academic stress. As a habit, psychoeducation is not enough for its modification. Instead, it requires developing skills to avoid the automatic activation of the habit and use alternative resources in these situations (Watkins and Roberts, 2020). The results contribute to the evidence of multifinality and divergence of RNT as a transdiagnostic process proposed by Nolen-Hoeksema and Watkins (2011). In this study, it was observed that reactive RNT was a predictor for most of the syndromes evaluated – somatization, obsessive-compulsive, interpersonal sensitivity, depression, paranoid ideation, psychoticism, and hostility – but it had no impact on the variation of anxiety symptoms, whose variation was due to daily stress. A high association between anxiety and stress had previously been established in the cognitive model of anxiety (Clark and Beck, 2011), although we also expected to observe the impact of worry (Ehring and Behar, 2020). Previous research suggests that the specific content of RNT, such as worrying, may be less important than the overall RNT process in contributing to anxiety (Spinhoven et al., 2018). This may also apply to our study.

In contrast, the development of symptoms of paranoia and interpersonal sensitivity, although influenced by RNT, was not affected by stress. A possible explanation is that they correspond to syndromes or characteristics that are more stable over time and less sensitive to stress. For example, interpersonal sensitivity is also conceived as a personality trait (Gillespie et al., 2001), while paranoia is considered both a symptom and a trait (Muñoz-Negro et al., 2019).

### 4.1 Limitations and future research implications

This study has several strengths. Firstly, it uses EMA to assess the levels of academic and non-academic stress and RNT that students experience in their daily lives, combined with traditional measures of thinking styles and baseline cumulative academic stress. Additionally, the study evaluated both the contents of the reactive RNT – brooding, reflection, and worry – and the elements of the RNT process, duration, and intrusion. Finally, the study provides evidence about transdiagnostic processes in the Spanish-speaking population, which is usually studied in English speakers (Hall et al., 2021). Despite these contributions, this research is not free of limitations. One of them is that the results of the predictive models of psychotic symptoms may have been affected because, at the beginning of the study, participants with high scores in psychoticism and suicidal risk were excluded. Although this decision is due to reasons of ethical care of the participants, it must be considered when interpreting these results. Another element to consider is that the seven-day follow-up period is relatively short, although it has allowed positive results in similar studies (Connolly and Alloy, 2017; Newman et al., 2019; Hjartarson et al., 2021, among others). Lastly, while the hierarchical regression analysis used in this study allowed us to determine the contributions of different groups of predictors to the development of symptoms, it did not enable us to evaluate interactions or chains of mediation between them. Future studies in the field would benefit from analysis with structural equation modeling (SEM) models such as those used by Smith et al. (2021). Using an EMA design and SEM analysis, this study evaluated the interaction between momentary rumination, negative affect, and binge eating.

The results achieved are helpful for the prevention of mental disorders in university contexts. Academic and non-academic stress in the students’ lives influenced the increase in symptoms of six of the eight syndromes evaluated. A recent systematic review of stress management programs for college students (Amanvermez et al., 2023), including 46 universal prevention studies, showed that universal stress management programs have moderate effects in reducing perceived stress and a large effect in reducing cortisol levels. Indicating that stress management programs can reduce stress and prevent mental health problems in higher education. In this sense, a previous systematic review concluded that universal preventive programs for university mental health problems with proven effectiveness are those with supervised skills training above psychoeducational or unsupervised training (Conley et al., 2015). Another element that emerges from this study is the effect of excessive use of RNT when facing university life stress. The need to intervene preventively for RNT in university students is even greater than in other age groups since recent longitudinal studies have determined that RNT is higher in young adulthood than at any other time in life (Lilly et al., 2023). Furthermore, another systematic review of selective and indicated preventive programs for college students shows the benefits of cognitive-behavioral programs, which often use a transdiagnostic approach (Barnett et al., 2021). In both cases, the reviews point to the convenience of acting preventively in this group, both to prevent mental disorders and to promote adequate adaptation and well-being in this period of life. The results of this study point to the development of preventive intervention programs for those young people with high RNT. In a recent study involving college students with high trait RNT, participants received an RNT-focused intervention (smartphone app-based, 10 days, 15 min per day). They were then exposed to a standardized psychosocial stressor. Those in the intervention group reported less negative affect during the recovery phase. Additionally, they rated their ability to cope with the stressor as higher and reported less RNT during the recovery phase after the stressor (Funk et al., 2024). In the case of university students needing treatment, targeting RNT becomes a necessary element. A qualitative study (Rogiers et al., 2022) of participants in group interventions aimed at managing repetitive negative thinking (PNR) identified several mechanisms perceived as effective: increasing awareness of PNR, experimenting with exposure rather than avoiding stressful situations, living in the “here and now,” thinking in concrete terms and moving from a “mode of thought” to a “mode of action.” Participants reported that these strategies were helpful tools, suggesting that they should be integral components of treatment approaches to coping with RNT. In this sense, there are new brief online interventions (with and without therapist guidance) to reduce RNT and symptoms of anxiety and depression that are achieving promising results (Joubert et al., 2023) and may be helpful for this group.

## 5 Conclusion

The stress of university life affects the development of various psychiatric symptoms in students. The research indicates that repetitive negative thinking is a transdiagnostic process in several syndromic groups beyond traditional emotional disorders. At the same time, it highlights the importance of the duration of the RNT process in developing symptoms. The study results suggest that universal preventive programs should consider the impact of academic and non-academic stress on university students’ mental health. Additionally, selective preventive interventions should focus on RNT to help students who are at risk of developing psychiatric symptoms.

## Data availability statement

The datasets presented in this article are not readily available because according to the informed consent forms completed by participants, the raw data collected during the study will remain confidential. Requests to access the datasets should be directed to clbustos@udec.cl.

## Ethics statement

The study was approved by the Scientific Ethical Committee of Universidad de Concepción (Certificate number CB1123-2022) and is in accordance with the Declaration of Helsinki. The studies were conducted in accordance with the local legislation and institutional requirements. The participants provided their written informed consent to participate in this study.

## Author contributions

CI: Conceptualization, Formal analysis, Funding acquisition, Investigation, Methodology, Project administration, Resources, Supervision, Validation, Writing – original draft, Writing – review & editing, Data curation. CB: Conceptualization, Formal analysis, Investigation, Methodology, Software, Supervision, Validation, Writing – original draft, Writing – review & editing. VB: Data curation, Investigation, Software, Visualization, Writing – review & editing. LG: Data curation, Investigation, Software, Visualization, Writing – review & editing. FC: Conceptualization, Data curation, Formal analysis, Methodology, Validation, Writing – original draft, Writing – review & editing.

## References

[ref1] Amanvermez Y. Rahmadiana M. Karyotaki E. de Wit L. Ebert D. D. Kessler R. C.. (2023). Stress management interventions for college students: a systematic review and meta-analysis. Clin. Psychol. Sci. Pract. 30, 423–444. doi: 10.1111/cpsp.12342

[ref2] Araneda-Guirriman C. Gairín J. Pedraja-Rejas L. Rodríguez-Ponce E. (2018). Percepciones sobre el perfil del estudiante universitario en el Contexto de la educación superior de masas: aproximaciones desde Chile. Interciencia 43, 864–870.

[ref3] Auerbach R. P. Mortier P. Bruffaerts R. Alonso J. Benjet C. Cuijpers P.. (2018). WHO world mental health surveys international college student project: prevalence and distribution of mental disorders. J. Abnorm. Psychol. 127, 623–638. doi: 10.1037/abn0000362, PMID: 30211576 PMC6193834

[ref4] Barlow D. H. Allen L. B. Choate M. L. (2004). Toward a unified treatment for emotional disorders. Behav. Ther. 35, 205–230. doi: 10.1016/S0005-7894(04)80036-4 27993336

[ref5] Barnett P. Arundell L. L. Saunders R. Matthews H. Pilling S. (2021). The efficacy of psychological interventions for the prevention and treatment of mental health disorders in university students: a systematic review and meta-analysis. J. Affect. Disord. 280, 381–406. doi: 10.1016/j.jad.2020.10.060, PMID: 33227669

[ref6] Barraza A. (2007). Propiedades psicométricas del Inventario SISCO del estrés académico. Rev. Psicol. 9, 40–50.

[ref7] Bravo A. J. Sotelo M. Pilatti A. Mezquita L. Read J. P. Cross-Cultural Addictions Study Team (2019). Depressive symptoms, ruminative thinking, marijuana use motives, and marijuana outcomes: a multiple mediation model among college students in five countries. Drug Alcohol Depend. 204:107558. doi: 10.1016/j.drugalcdep.2019.107558, PMID: 31586807 PMC6878192

[ref8] Carrozzino D. Patierno C. Guidi J. Berrocal Montiel C. Cao J. Charlson M. E.. (2021). Clinimetric criteria for patient-reported outcome measures. Psychother. Psychosom. 90, 222–232. doi: 10.1159/000516599 34038901

[ref9] Castillo-Navarrete J. L. Guzmán-Castillo A. Bustos C. Zavala W. Vicente B. (2020). Propiedades psicométricas del inventario SISCO-II de estrés académico. *Revista Iberoamericana de Diagnóstico y Evaluación – e Avaliação Psicológica*. RIDEP 56, 101–116. doi: 10.21865/RIDEP56.3.08

[ref10] Clark D. A. Beck A. T. (2011). Cognitive therapy of anxiety disorders: Science and practice. New York: Guilford Press.

[ref11] Conley C. S. Durlak J. A. Kirsch A. C. (2015). A Meta-analysis of universal mental health prevention programs for higher education students. Prev. Sci. 16, 487–507. doi: 10.1007/s11121-015-0543-1, PMID: 25744536

[ref12] Connolly S. L. Alloy L. B. (2017). Rumination interacts with life stress to predict depressive symptoms: an ecological momentary assessment study. Behav. Res. Ther. 97, 86–95. doi: 10.1016/j.brat.2017.07.006, PMID: 28734979 PMC5600682

[ref13] Cova F. Garcia F. Oyanadel C. Villagran L. Páez D. Inostroza C. (2019). Adaptive reflection on negative emotional experiences: convergences and divergence between the processing-mode theory and the theory of self-distancing reflection. Front. Psychol. 10:1943. doi: 10.3389/fpsyg.2019.01943, PMID: 31551854 PMC6734160

[ref14] Cova F. Melipillán R. Rincón P. (2007). Rumiación y presencia de sintomatología ansiosa y depresiva en adolescentes. Rev. Mexic. Psicol. 24, 175–183. doi: 10.4067/S0718-48082007000200001

[ref15] Cova F. Melipillán R. Rincón P. (2009). Reflexión, rumiación negativa y desarrollo de sintomatología depresiva en adolescentes de sexo femenino. Terapia Psicol. 27, 155–160. doi: 10.4067/S0718-48082009000200001

[ref16] Cranford J. A. Shrout P. E. Iida M. Rafaeli E. Yip T. Bolger N. (2006). A procedure for evaluating sensitivity to within-person change: can mood measures in diary studies detect change reliably? Personal. Soc. Psychol. Bull. 32, 917–929. doi: 10.1177/0146167206287721, PMID: 16738025 PMC2414486

[ref17] Dalgleish T. Black M. Johnston D. Bevan A. (2020). Transdiagnostic approaches to mental health problems: current status and future directions. J. Consult. Clin. Psychol. 88, 179–195. doi: 10.1037/ccp0000482, PMID: 32068421 PMC7027356

[ref18] Derogatis L. R. (1975). SCL-90-R. Baltimore, MD: Johns Hopkins University.

[ref19] Ehring T. Behar E. (2020). Transdiagnostic view on worrying and other negative mental content. Gene. Anxiety Disord. Worry. 43-68. doi: 10.1002/9781119189909.ch4

[ref20] Ehring T. Watkins E. R. (2008). Repetitive negative thinking as a transdiagnostic process. Int. J. Cogn. Ther. 1, 192–205. doi: 10.1521/ijct.2008.1.3.192

[ref21] Ehring T. Zetsche U. Weidacker K. Wahl K. Schönfeld S. Ehlers A. (2011). The perseverative thinking questionnaire (PTQ): validation of a content-independent measure of repetitive negative thinking. J. Behav. Ther. Exp. Psychiatry 42, 225–232. doi: 10.1016/j.jbtep.2010.12.003, PMID: 21315886 PMC3042595

[ref22] Eustis E. H. Cardona N. Nauphal M. Sauer-Zavala S. Rosellini A. J. Farchione T. J.. (2020). Experiential avoidance as a mechanism of change across cognitive-behavioral therapy in a sample of participants with heterogeneous anxiety disorders. Cogn. Ther. Res. 44, 275–286. doi: 10.1007/s10608-019-10063-6

[ref23] Evans-Lacko S. Thornicroft G. (2019). WHO world mental health surveys international college student initiative: implementation issues in low-and middle-income countries. Int. J. Methods Psychiatr. Res. 28:e1756. doi: 10.1002/mpr.1756, PMID: 30614124 PMC6877214

[ref24] Ferrer L. Martin-Vivar M. Pineda D. Sandin B. Piqueras J. A. (2018). Relationship of anxiety and depressive symptomatology in adolescents with two transdiagnostic mechanisms: perfectionism and rumination. Psicol. Conduct. 26, 55–74.

[ref25] Funk J. Takano K. Babl M. Goldstein R. Oberwestersberger R. Kopf-Beck J.. (2024). Can an intervention designed to reduce repetitive negative thinking alter the response to a psychosocial stressor? A randomized controlled study. Behav. Res. Ther. 178:104547. doi: 10.1016/j.brat.2024.104547, PMID: 38678755

[ref9001] Gardani M. Bradford D. R. Russell K. Allan S. Beattie L. Ellis J. G. (2022). A systematic review and meta-analysis of poor sleep, insomnia symptoms and stress in undergraduate students. Sleep medicine reviews 61:101565. doi: 10.1016/j.smrv.2021.101565 34922108

[ref26] Gempp Fuentealba R. Avendaño Bravo C. (2008). Datos normativos y propiedades psicométricas del SCL-90-R en estudiantes universitarios chilenos. Terap. Psicol. 26, 39–58. doi: 10.4067/S0718-48082008000100004

[ref27] Gillespie N. A. Johnstone S. J. Boyce P. Heath A. C. Martin N. G. (2001). The genetic and environmental relationship between the interpersonal sensitivity measure (IPSM) and the personality dimensions of Eysenck and Cloninger. Personal. Individ. Differ. 31, 1039–1051. doi: 10.1016/S0191-8869(00)00200-2

[ref28] Gustavson K. Knudsen A. K. Nesvåg R. Knudsen G. P. Vollset S. E. Reichborn-Kjennerud T. (2018). Prevalence and stability of mental disorders among young adults: findings from a longitudinal study. BMC Psychiatry 18, 65–15. doi: 10.1186/s12888-018-1647-5, PMID: 29530018 PMC5848432

[ref29] Guzmán-Castillo A. Bustos C. Zavala W. Castillo-Navarrete J. L. (2022). Inventario SISCO del estrés académico: revisión de sus propiedades psicométricas en estudiantes universitarios. Terap. Psicol. 40, 197–211. doi: 10.4067/S0718-48082022000200197

[ref30] Hall M. Scherner P. V. Kreidel Y. Rubel J. A. (2021). A systematic review of momentary assessment designs for mood and anxiety symptoms. Front. Psychol. 12:642044. doi: 10.3389/fpsyg.2021.642044, PMID: 34079492 PMC8165285

[ref31] Hamonniere T. Laqueille X. Vorspan F. Dereux A. Illel K. Varescon I. (2020). Toward a better understanding of the influence of repetitive negative thinking in alcohol use disorder: an examination of moderation effect of metacognitive beliefs and gender. Addict. Behav. 111:106561. doi: 10.1016/j.addbeh.2020.106561, PMID: 32739590

[ref32] Harvey A. G. Watkins E. R. Mansell W. Shafran R. (2004). Cognitive behavioural processes across psychological disorders: A transdiagnostic approach to research and treatment. Oxford: Oxford University Press.

[ref33] Heckendorf H. Lehr D. Ebert D. D. Freund H. (2019). Efficacy of an internet and app-based gratitude intervention in reducing repetitive negative thinking and mechanisms of change in the intervention's effect on anxiety and depression: results from a randomized controlled trial. Behav. Res. Ther. 119:103415. doi: 10.1016/j.brat.2019.103415, PMID: 31202003

[ref34] Hijne K. Penninx B. W. van Hemert A. M. Spinhoven P. (2020). The association of changes in repetitive negative thinking with changes in depression and anxiety. J. Affect. Disord. 275, 157–164. doi: 10.1016/j.jad.2020.07.002, PMID: 32734902

[ref35] Hjartarson K. H. Snorrason I. Bringmann L. F. Ögmundsson B. E. Ólafsson R. P. (2021). Do daily mood fluctuations activate ruminative thoughts as a mental habit? Results from an ecological momentary assessment study. Behav. Res. Ther. 140:103832. doi: 10.1016/j.brat.2021.103832, PMID: 33765651

[ref36] Joubert A. E. Grierson A. B. Li I. Sharrock M. J. Moulds M. L. Werner-Seidler A.. (2023). Managing rumination and worry: a randomised controlled trial of an internet intervention targeting repetitive negative thinking delivered with and without clinician guidance. Behav. Res. Ther. 168:104378. doi: 10.1016/j.brat.2023.104378, PMID: 37595354

[ref9002] Jurado-Botina L. Montero-Bolaños C. Carlosama-Rodríguez D. Tabares-Díaz Y. (2021). Estrés académico en estudiantes universitarios de Iberoamérica: una revisión sistemática. Cuadernos hispanoamericanos de psicología 2021, 1–18. doi: 10.18270/chps.v2021i2.3917

[ref37] Karyotaki E. Cuijpers P. Albor Y. Alonso J. Auerbach R. P. Bantjes J.. (2020). Sources of stress and their associations with mental disorders among college students: results of the world health organization world mental health surveys international college student initiative. Front. Psychol. 11:1759. doi: 10.3389/fpsyg.2020.01759, PMID: 32849042 PMC7406671

[ref38] Klemanski D. H. Curtiss J. McLaughlin K. A. Nolen-Hoeksema S. (2017). Emotion regulation and the transdiagnostic role of repetitive negative thinking in adolescents with social anxiety and depression. Cogn. Ther. Res. 41, 206–219. doi: 10.1007/s10608-016-9817-6, PMID: 28579659 PMC5455341

[ref39] Kummer K. Mattes A. Stahl J. (2024). Do perfectionists show negative, repetitive thoughts facing uncertain situations? Curr. Psychol. 43, 2387–2402. doi: 10.1007/s12144-023-04409-3

[ref40] Lawrence A. V. Alkozei A. Irgens M. S. Acevedo-Molina M. C. Brener S. A. Chandler A. B.. (2021). Think again: adaptive repetitive thought as a Transdiagnostic treatment for individuals predisposed to repetitive thinking styles. J. Psychother. Integr. 31, 208–222. doi: 10.1037/int0000209

[ref41] Leonardo C. Aristide S. Michela B. (2021). On the efficacy of the Barlow unified protocol for Transdiagnostic treatment of emotional disorders: a systematic review and meta-analysis. Clin. Psychol. Rev. 87:101999. doi: 10.1016/j.cpr.2021.101999 34098412

[ref42] Liew J. Morris A. S. Kerr K. L. (2023). “Emotion regulation processes as transdiagnostic in adolescence and early adulthood: a neurobioecological systems framework” in APA handbook of adolescent and young adult development. eds. Crockett L. J. Carlo G. Schulenberg J. E. (Washington, DC: American Psychological Association), 91–106. doi: 10.1037/0000298-006

[ref43] Lignier B. Petot J. Canada B. Nicolas M. Courtois R. De Oliveira P. (2024). The structure of the symptom Checklist-90-revised: global distress, somatization, hostility, and phobic anxiety scales are reliable and robust across community and clinical samples from four European countries. Psychiatry Res. 331:115635. doi: 10.1016/j.psychres.2023.115635, PMID: 38101071

[ref44] Lilly K. J. Howard C. Zubielevitch E. Sibley C. G. (2023). Thinking twice: examining gender differences in repetitive negative thinking across the adult lifespan. Front. Psychol. 14:1239112. doi: 10.3389/fpsyg.2023.1239112, PMID: 38022916 PMC10663279

[ref45] Liu Y. De A. (2015). Multiple imputation by fully conditional specification for dealing with missing data in a large epidemiologic study. Int. J. Statist. Med. Res. 4, 287–295. doi: 10.6000/1929-6029.2015.04.03.7, PMID: 27429686 PMC4945131

[ref46] Luca M. (2019). Maladaptive rumination as a transdiagnostic mediator of vulnerability and outcome in psychopathology. J. Clin. Med. 8:314. doi: 10.3390/jcm8030314, PMID: 30841644 PMC6463018

[ref47] Mac-Ginty S. Jiménez-Molina A. Martínez V. (2021). Impacto de la pandemia por COVID-19 en la salud mental de estudiantes universitarios en Chile. Rev. Chilena Psiquiatría Neurol. Infanc. Adolesc. 32, 23–37.

[ref48] Mansueto G. Cavallo C. Palmieri S. Ruggiero G. M. Sassaroli S. Caselli G. (2021). Adverse childhood experiences and repetitive negative thinking in adulthood: a systematic review. Clin. Psychol. Psychother. 28, 557–568. doi: 10.1002/cpp.2590, PMID: 33861493

[ref49] Mansueto G. Jarach A. Caselli G. Ruggiero G. M. Sassaroli S. Nikčević A.. (2024). A systematic review of the relationship between generic and specific metacognitive beliefs and emotion dysregulation: a metacognitive model of emotion dysregulation. Clin. Psychol. Psychother. 31:e2961. doi: 10.1002/cpp.2961, PMID: 38357852

[ref50] Mansueto G. Marino C. Palmieri S. Offredi A. Sarracino D. Sassaroli S.. (2022). Difficulties in emotion regulation: the role of repetitive negative thinking and metacognitive beliefs. J. Affect. Disord. 308, 473–483. doi: 10.1016/j.jad.2022.04.086, PMID: 35460736

[ref51] Martínez P. Jiménez-Molina Á. Mac-Ginty S. Martínez V. Rojas G. (2021). Salud mental en estudiantes de educación superior en Chile: una revisión de alcance con meta-análisis. Terap. Psicol. 39, 405–426. doi: 10.4067/S0718-48082021000300405

[ref52] Mathes B. M. Kennedy G. A. Morabito D. M. Martin A. Bedford C. E. Schmidt N. B. (2020). A longitudinal investigation of the association between rumination, hostility, and PTSD symptoms among trauma-exposed individuals. J. Affect. Disord. 277, 322–328. doi: 10.1016/j.jad.2020.08.029, PMID: 32858313

[ref53] Meyer T. Miller M. Metzger R. Borkovec T. (1990). Development and validation of the Penn State worry questionnaire. Behav. Res. Ther. 28, 487–495. doi: 10.1016/0005-7967(90)90135-6 2076086

[ref54] Micin S. Bagladi V. (2011). Salud Mental en Estudiantes Universitarios: Incidencia de Psicopatología y Antecedentes de Conducta Suicida en Población que Acude a un Servicio de Salud Estudiantil. Terap. Psicol. 29, 53–64. doi: 10.4067/S0718-48082011000100006

[ref55] Miethe S. Muehlhan M. Trautmann S. (2022). The association between repetitive negative thinking and distress across mental disorders: preliminary findings from an outpatient treatment-seeking sample. Psychiatry Res. 311:114478. doi: 10.1016/j.psychres.2022.114478, PMID: 35287044

[ref56] Miranda R. Nolen-Hoeksema S. (2007). Brooding and reflection: rumination predicts suicidal ideation at 1-year follow-up in a community sample. Behav. Res. Ther. 45, 3088–3095. doi: 10.1016/j.brat.2007.07.015, PMID: 17825248 PMC4026259

[ref57] Muñoz-Negro J. E. Prudent C. Gutiérrez B. Cervilla J. A. (2019). Paranoia and risk of personality disorder in the general population. Personal. Ment. Health 13, 107–116. doi: 10.1002/pmh.1443, PMID: 30989831

[ref58] Neshat Z. Farah Bijari A. Dehshiri G. (2024). The relationship between perfectionism and interpersonal sensitivity with self-compassion in university students: the mediation of repetitive negative thinking. Psychol. Beiträge 25, 107–120. doi: 10.1007/s10339-023-01163-z, PMID: 37803210

[ref59] Newman M. G. Jacobson N. C. Zainal N. H. Shin K. E. Szkodny L. E. Sliwinski M. J. (2019). The effects of worry in daily life: an ecological momentary assessment study supporting the tenets of the contrast avoidance model. Clin. Psychol. Sci. 7, 794–810. doi: 10.1177/2167702619827019, PMID: 31372313 PMC6675025

[ref60] Newman M. G. Llera S. J. Erickson T. M. Przeworski A. Castonguay L. G. (2013). Worry and generalized anxiety disorder: a review and theoretical synthesis of evidence on nature, etiology, mechanisms, and treatment. Annu. Rev. Clin. Psychol. 9, 275–297. doi: 10.1146/annurev-clinpsy-050212-185544, PMID: 23537486 PMC4964851

[ref61] Nolen-Hoeksema S. (1991). Responses to depression and their effects on the duration of depressive episodes. J. Abnorm. Psychol. 100, 569–582. doi: 10.1037/0021-843X.100.4.569 1757671

[ref62] Nolen-Hoeksema S. Watkins E. R. (2011). A heuristic for developing transdiagnostic models of psychopathology: explaining multifinality and divergent trajectories. Perspect. Psychol. Sci. 6, 589–609. doi: 10.1177/1745691611419672, PMID: 26168379

[ref63] Nolen-Hoeksema S. Wisco B. E. Lyubomirsky S. (2008). Rethinking rumination. Perspect. Psychol. Sci. 3, 400–424. doi: 10.1111/j.1745-6924.2008.00088.x 26158958

[ref64] Norton P. J. Paulus D. J. (2016). Toward a unified treatment for emotional disorders: update on the science and practice. Behav. Ther. 47, 854–868. doi: 10.1016/j.beth.2015.07.002, PMID: 27993337

[ref65] Ochnik D. Rogowska A. M. Kuśnierz C. Jakubiak M. Schütz A. Held M. J.. (2021). Mental health prevalence and predictors among university students in nine countries during the COVID-19 pandemic: a cross-national study. Sci. Rep. 11:18644 (2021). doi: 10.1038/s41598-021-97697-3, PMID: 34545120 PMC8452732

[ref66] Palmieri S. Mansueto G. Scaini S. Caselli G. Sapuppo W. Spada M. M.. (2021). Repetitive negative thinking and eating disorders: a meta-analysis of the role of worry and rumination. J. Clin. Med. 10:2448. doi: 10.3390/jcm10112448, PMID: 34073087 PMC8198834

[ref67] Palmieri S. Mansueto G. Scaini S. Fiore F. Sassaroli S. Ruggiero G. M.. (2018). Role of rumination in the relationship between metacognition and shyness. World J. Psychiatry 8, 108–113. doi: 10.5498/wjp.v8.i4.108, PMID: 30370229 PMC6201322

[ref68] Palmieri S. Sassaroli S. Ruggiero G. M. Caselli G. Nocita R. Nikčević A.. (2024). Perfectionism in patients with eating disorders: the role of metacognitive beliefs and repetitive negative thinking. Clin. Psychol. Psychother. 31:e2954. doi: 10.1002/cpp.2954 42053044

[ref69] Palmieri S. Sassaroli S. Ruggiero G. M. Caselli G. Spada M. M. Mansueto G. (2023). Emotion dysregulation in patients with eating disorders: the role of metacognitions and repetitive negative thinking. Cogn. Ther. Res. 47, 655–668. doi: 10.1007/s10608-023-10398-1

[ref70] Papageorgiou C. (2006). Worry and rumination: Styles of persistent negative thinking in anxiety and depression, in Worry and its psychological disorders: Theory, assessment and treatment. (Eds.) Davey G. C. L. Wells A., (Hoboken, NJ: Wiley Publishing), 21–40.

[ref90001] Pascual-Vera B. Belloch A. (2018). El carácter transdiagnóstico de las intrusiones mentales: Una revisión y una propuesta basada en datos [The transdiagnostic nature of mental intrusions: A review and data-based proposal]. Revista de Psicopatología y Psicología Clínica. 23, 135–147. doi: 10.5944/rppc.vol.23.num.2.2018.20738

[ref71] Pimentel M. Cova F. (2011). Efectos de la rumiación y la preocupación en el desarrollo de sintomatología depresiva y ansiosa en estudiantes universitarios de la ciudad de Concepción, Chile. Terap. Psicol. 29, 43–52. doi: 10.4067/s0718-48082011000100005

[ref72] Pitt A. Oprescu F. Tapia G. Gray M. (2018). An exploratory study of students’ weekly stress levels and sources of stress during the semester. Act. Learn. High. Educ. 19, 61–75. doi: 10.1177/1469787417731194

[ref73] Rodríguez R. Vetere G. (2006). Adaptación Argentina del Cuestionario de Preocupaciones de Pensilvania: resultados preliminares sobre su validez y confiabilidad. XIII Jornadas de Investigación y Segundo Encuentro de Investigadores en Psicología del Mercosur. Buenos Aires: Facultad de Psicología-Universidad de Buenos Aires.

[ref74] Rogers M. L. Gallyer A. J. Joiner T. E. (2021). The relationship between suicide-specific rumination and suicidal intent above and beyond suicidal ideation and other suicide risk factors: a multilevel modeling approach. J. Psychiatr. Res. 137, 506–513. doi: 10.1016/j.jpsychires.2021.03.031 33812323

[ref75] Rogiers R. Van Parys H. Baeken C. Van den Abbeele D. Remue J. De Raedt R.. (2022). Treatment experiences during a cognitive behaviour therapy group intervention targeting repetitive negative thinking: a qualitative study. Psychol. Psychother. Theory Res. Pract. 95, 447–466. doi: 10.1111/papt.12378, PMID: 34936174

[ref76] Rosenkranz T. Takano K. Watkins E. R. Ehring T. (2020). Assessing repetitive negative thinking in daily life: development of an ecological momentary assessment paradigm. PLoS One 15:e0231783. doi: 10.1371/journal.pone.0231783, PMID: 32310979 PMC7170251

[ref77] Rossi J. L. Jiménez J. P. Barros P. Assar R. Jaramillo K. Herrera L.. (2019). Sintomatología depresiva y bienestar psicológico en estudiantes universitarios chilenos. Rev. Med. Chile 147, 579–588. doi: 10.4067/S0034-98872019000500579, PMID: 31859890

[ref78] Saldivia S. Aslan-Parra J. Bustos C. Inostroza C. Cova F. Castillo A. (2023). Latent profiles for mental health in older people from Concepción, Chile. Eur. Psychiatry 66:S235. doi: 10.1192/j.eurpsy.2023.542

[ref9004] Sandín B. Chorot P. Valiente R. M. (2012). Transdiagnóstico: nueva frontera en psicología clínica. Revista de psicopatología y psicología clínica, 17.

[ref79] Sandín B. Chorot P. Valiente R. M. Lostao L. (2009). Validación española del cuestionario de preocupación PSWQ: Estructura factorial y propiedades psicométricas. Rev. Psicopatol. Psicol. Clín. 14, 107–122. doi: 10.5944/rppc.vol.14.num.2.2009.4070

[ref80] Sheldon E. Simmonds-Buckley M. Bone C. Mascarenhas T. Chan N. Wincott M.. (2021). Prevalence and risk factors for mental health problems in university undergraduate students: a systematic review with meta-analysis. J. Affect. Disord. 287, 282–292. doi: 10.1016/j.jad.2021.03.054, PMID: 33812241

[ref81] Shiffman S. Stone A. A. Hufford M. R. (2008). Ecological momentary assessment. Annu. Rev. Clin. Psychol. 4, 1–32. doi: 10.1146/annurev.clinpsy.3.022806.091415 18509902

[ref82] Smith K. E. Mason T. B. Reilly E. E. Hazzard V. M. Borg S. L. Dvorak R.. (2021). Examining prospective mediational relationships between momentary rumination, negative affect, and binge eating using ecological momentary assessment. J. Affect. Disord. Rep. 5:100138. doi: 10.1016/j.jadr.2021.100138, PMID: 34458883 PMC8388245

[ref83] Spinhoven P. van Hemert A. M. Penninx B. W. (2018). Repetitive negative thinking as a predictor of depression and anxiety: a longitudinal cohort study. J. Affect. Disord. 241, 216–225. doi: 10.1016/j.jad.2018.08.037, PMID: 30138805

[ref84] Spinhoven P. van Hemert A. M. Penninx B. W. (2019). Repetitive negative thinking as a mediator in prospective cross-disorder associations between anxiety and depression disorders and their symptoms. J. Behav. Ther. Exp. Psychiatry 63, 6–11. doi: 10.1016/j.jbtep.2018.11.007 30551055

[ref85] Treynor W. Gonzalez R. Nolen-Hoeksema S. (2003). Rumination reconsidered: a psychometric analysis. Cogn. Ther. Res. 27, 247–259. doi: 10.1023/A:1023910315561

[ref86] Tucker R. P. Wingate L. R. O’Keefe V. M. Mills A. C. Rasmussen K. Davidson C. L.. (2013). Rumination and suicidal ideation: the moderating roles of hope and optimism. Personal. Individ. Differ. 55, 606–611. doi: 10.1016/j.paid.2013.05.013

[ref87] Turecki G. Brent D. A. Gunnell D. O’Connor R. C. Oquendo M. A. Pirkis J.. (2019). Suicide and suicide risk. Nat. Rev. Dis. Prim. 5, 1–22. doi: 10.1038/s41572-019-0121-0 31649257

[ref88] Valencia P. D. (2020). Initial psychometric evaluation of the perseverative thinking questionnaire in Peruvian undergraduates. Liberabit 26:e404. doi: 10.24265/liberabit.2020.v26n2.05

[ref89] Vrieze S. (2012). Model selection and psychological theory: a discussion of the differences between the Akaike information criterion (AIC) and the Bayesian information criterion (BIC). Psychol. Methods 17, 228–243. doi: 10.1037/a0027127 22309957 PMC3366160

[ref90] Wahl K. Ehring T. Kley H. Lieb R. Meyer A. Kordon A.. (2019). Is repetitive negative thinking a transdiagnostic process? A comparison of key processes of RNT in depression, generalized anxiety disorder, obsessive-compulsive disorder, and community controls. J. Behav. Ther. Exp. Psychiatry 64, 45–53. doi: 10.1016/j.jbtep.2019.02.006, PMID: 30851652

[ref91] Watkins E. R. Roberts H. (2020). Reflecting on rumination: consequences, causes, mechanisms and treatment of rumination. Behav. Res. Ther. 127:103573. doi: 10.1016/j.brat.2020.103573, PMID: 32087393

[ref9005] Watkins E. R. (2008). Constructive and unconstructive repetitive thought. Psychological bulletin, 134:163. doi: 10.1037/0033-2909.134.2.163 18298268 PMC2672052

[ref92] Wong Q. J. McEvoy P. M. Rapee R. M. (2019). Repetitive thinking in social anxiety disorder: are anticipatory processing and post-event processing facets of an underlying unidimensional construct? Behav. Ther. 50, 571–581. doi: 10.1016/j.beth.2018.09.004, PMID: 31030874

[ref93] World Health Organization (2014). Preventing suicide: A global imperative. Geneva: World Health Organization.

[ref94] Zagaria A. Ballesio A. Vacca M. Lombardo C. (2023). Repetitive negative thinking as a central node between psychopathological domains: a network analysis. Int. J. Cogn. Ther. 16, 143–160. doi: 10.1007/s41811-023-00162-4

